# Synergistic effects of air pollution and temperature on blood pressure in older German women

**DOI:** 10.1038/s41598-026-51334-z

**Published:** 2026-05-13

**Authors:** Dayasri Ravi, Andreas Groll, Claudia Wigmann, Nidhi Singh, Tamara Schikowski

**Affiliations:** 1 Department of Statistics, TU Dortmund University, Vogelpothsweg 87, 44227 Dortmund, NRW Germany; 2 IUF-Leibniz Research Institute for Environmental Medicine, Auf’m Hennekamp 50, 40225 Düsseldorf, NRW Germany; 3 Department of Environment and Health, School of Public Health, University of Bielefeld, 33501 Bielefeld, Germany

**Keywords:** Air pollution, Temperature, Blood pressure, Simultaneous exposure, Linear and non-linear interactive effects, Cardiology, Environmental sciences, Environmental impact

## Abstract

Studies have shown how air pollution and temperature affect blood pressure. We investigated the combined effect of air pollution and temperature on blood pressure in a cohort of older German women. We conducted a cross-sectional analysis of systolic (SBP) and diastolic blood pressure (DBP) data from follow-up 3 (2012–2013) of the Study on the influence of Air pollution on lung function inflammation and ageing (SALIA) cohort. Short-term data on daily air pollutants and temperature were obtained from the German Weather Bureau and the German Environment Agency. A generalized additive model was used to capture their combined effect. Stratified analyses were performed to quantify the variation in the estimated effects. The core model was adjusted for potential covariates. We observed a combined association of temperature and air pollution with blood pressure. We found that lower temperatures and higher levels of air pollutants such as PM_2.5_ and NO_2_ were associated with higher SBP and DBP. However, higher O_3_ exposure was generally associated with lower SBP and DBP. Stratified analyses showed that the associations between temperature, air pollution, and blood pressure were stronger among women living in urban areas and those with lower socio-economic status. The combined effect of low ambient temperature and high air pollution substantially increased BP among older German women.

**Supplementary Information:**

The online version contains supplementary material available at 10.1038/s41598-026-51334-z.

## Introduction

Every year, an estimated seven million premature deaths are attributed to the combined effects of household and ambient air pollution^1^. There is substantial and consistent evidence, from studies conducted over both short and long periods, that air pollution is associated with increased mortality and morbidity risks related to hypertension, cardiovascular disease, respiratory conditions, and stroke, as well as more frequent emergency visits for hypertension^2,3^. Possible explanations for this association include elevated oxidative stress levels, systemic inflammation, endothelial dysfunction, alterations in blood coagulability, and the autonomic nervous system^4,5^. Similarly, extreme temperatures have been associated with variations in blood pressure (BP), leading to more cardiovascular deaths^6,7^. However, the current evidence is drawn from studies across varied geographic regions and age groups, resulting in inconsistent findings.

One of the reasons why prior studies have failed to provide conclusive evidence is the inability to account for the synergistic interactions between environmental exposures. Although the independent effects of temperature and outdoor pollutants on health are well-established, little effort has been made to explore their combined effects. The relationship between temperature and air pollution is influenced by factors such as location, climate, emissions, and specific pollutants. High temperatures can alter chemical reactions in the atmosphere, especially those involving nitrogen oxides^8–10^. On hot, sunny days, tropospheric ozone and other secondary pollutants often exceed normal levels. This combination of elevated temperatures and ozone can affect the body’s thermoregulation, potentially leading to decreased BP^11^. Therefore, understanding how air quality interacts with temperature and impacts health is critical. Recent epidemiological research emphasizes the urgency of investigating the health impacts of these environmental interactions^12^.

To address this research gap, we hypothesized a complex interaction, defined as a non-linear and non-additive relationship, between temperature and air pollution and their effects on BP in older females. Evidence suggests that older adults are more vulnerable to the harmful impacts of climate change^13^. Therefore, we utilized the well-characterized SALIA (Study on Air pollution, Lung function, Inflammation, and Aging) cohort to examine the association between temperature, air pollution and BP and conduct subgroup analyses to identify vulnerable populations to environmental exposures. Instead of using simple multiplication as in previous studies^8^, we employed a bivariate tensor product approach to capture these non-linear and non-additive exposure–response relationships between environmental stressors and the BP. Our research utilized generalized additive models (GAMs)^14^ with tensor product terms to accurately describe the complex interplay between temperature, air pollutants, and BP. These flexible models can capture non-linearities and incorporate multiple factors, demonstrating superior predictive power over linear models for assessing environmental health impacts. Thus, our study is novel in its application of robust statistical techniques like GAMs with a bivariate tensor product to model the combined effects of temperature and air pollution on BP.

## Material and methods

### Study design and population

The SALIA cohort study was established between 1985 and 1994 to investigate the health effects of air pollution exposure in women by the State Government of North-Rhine Westphalia, Germany. Women from the region’s industrialized Ruhr Area (Dortmund, Duisburg, Herne, Gelsenkirchen, and Essen) and two non-industrialized rural communities north of the Ruhr Area (Borken and Dülmen) make up the study population. The details of the study have been published elsewhere^15,16^. In the present cross-sectional study, we used data from follow-up 3 (2012–2013), which initially included 624 women. Participants were excluded if information on key covariates or outcome variables was missing. After excluding individuals with incomplete data, the final analytical sample consisted of 530 women with complete information on SBP, DBP and all model covariates. The exclusions were based solely on data completeness and were unlikely to have substantially affected the results. The study was approved by the ethics committee of the Medical Faculty of the Heinrich Heine University Düsseldorf (Germany; Registration number: 3507). All methods were performed in accordance with the relevant guidelines. The Declaration of Helsinki Principles was followed, and all women gave their written informed consent before the investigation.

### Assessment of BP and other covariates

During the third follow-up, participants were interviewed about their medical history, including diabetes mellitus, respiratory health conditions, cardiovascular diseases, medication, and lifestyle factors, following the standard study protocol. A sphygmomanometer, Omron 705 IT, at the left upper arm measured BP at a sitting position after a rest period of at least 5 min. The final BP value was, as a rule, defined as the mean value of the second and third measurements. Systolic (SBP) and diastolic BP (DBP) were used as BP markers.

We obtained information about other potentially confounding factors, such as smoking status, current passive smoking at home, the pack-years (number of cigarette packs consumed per day times the duration of smoking in years), urban/rural living, age, and body mass index (BMI). Socio-economic status (SES) was defined using the highest level of education attained within the household, categorized as < 10 years, 10 years, or > 10 years of schooling. This composite measure was used to reflect household-level socio-economic resources, consistent with established practice in cohort studies of older populations. The participants were classified as physically active or inactive based on self-reported regular participation in sports. Additionally, the frequency of alcohol consumption was used as a dichotomized variable (less than once a week vs. once a week or more often). Information on diabetes was included in sensitivity analyses.

### Exposure assessment

Individual exposure to short-term ambient air pollution and mean temperature was estimated at women’s residential addresses. In this study, we assigned exposure data at lag 0 (same day as BP measurement).

The daily mean temperatures (Tmean) and relative humidity (RH) were obtained from COSMO-REA6^17^ at a spatial resolution of 6 × 6 km for the study region.

We obtained data for daily air pollution levels (PM_2.5,_ NO_2_, and O_3_) at a spatial resolution of 2 × 2 km based on the method of optimal interpolation from the German Environment Agency^18^. The daily averages comprise 24-h mean exposure starting from midnight of the day of examination.

### Statistical analysis

We used GAM with an identity link to analyze the association between different BP measurements (SBP or DBP), Tmean, and air pollutants (PM_2.5_, NO_2_, or O_3_). The BP measurements were log-transformed to normalize the data and stabilize the variance. To rule out potential confounding, all models were adjusted for age, BMI, residential location (urban vs. rural), SES (low, medium, high), fossil fuel heating (yes/no), cumulative smoking exposure measured in pack-years, current smoking status, and passive smoking exposure. Additionally, all models included the season (warm: April-September, cold: October–March) as a binary temporal variable. Finally, all models contained an indicator variable for RH greater than 80%.

We investigated the combined effect of Tmean and air pollutants on BP through bivariate tensor product penalized splines. Therefore, we modeled BP as a bivariate function of Tmean and one air pollutant at a time. Non-linear smooth terms were specified for age and pack-years, while all other covariates entered the model parametrically.

$$\begin{aligned} \log E\left[ {Y|x} \right] = & \,te\left( {Tmean,PM_{2.5} /NO_{2} /O_{3} } \right) + s\left( {age} \right) + s\left( {packyears} \right) + BMI \\ & \, + location + SES + RH_{bin} + fossil\,heating + current\,smoker + passive\,smoking + season \\ \end{aligned}$$

where Y represents the BP with expectation *E*[Y|x] (conditional on all covariates x), *te*(*·*) is the bivariate tensor product of Tmean and one of the air pollutants, and *s*(*·*) symbolizes the (smooth) non-linear covariate effects based on penalized splines.

To evaluate seasonal effect modification, we extended the core GAM by allowing the bivariate tensor product smooth of temperature and air pollution to differ between cold and warm seasons through an interaction effect with the seasonal indicator. Seasonal modification was quantified by comparing the predicted joint exposure–response surfaces for warm versus cold seasons while holding all other covariates constant.

We performed several sensitivity analyses to test the robustness of the core model. We repeated the main analysis without applying the log transformation of BP. We additionally adjusted for frequency of alcohol intake, physical activity, and diabetes in the main model.

Furthermore, we examined effect modifications based on stratified analyses using individual characteristics such as location, BMI, and SES.

The strength of the non-linearity of GAM estimates was tested through both generalized cross-validation (GCV) scores and restricted maximum likelihood estimation (REML).

We also explored potential delayed effects by considering moving average exposure windows of 0 to 1 day and 0 to 3 days. Lag 0 to 3 was defined as the moving average of Tmean and each air pollutant from the day of the blood pressure examination through the three preceding days.

All analyses were performed using R statistical software, V4.3.3^19^.

## Results

### Descriptive results

Detailed characteristics of the study population are shown in Table 1. The average age of the study participants was 77.5 (± 3.15). Most of the participants had a higher BMI above 25 (79.4%) (Table S6), medium to high SES (81.3%), and were non-smokers (96.9%). The proportions of participants from urban and rural areas were relatively similar.

**Table 1:** Descriptive statistics of the study population.

	Overall (*N* = 530)
Age in years	
Mean (SD)	77.5 (3.15)
Median [Min, Max]	77.5 [70.1, 83.4]
BMI in kg/m^2^	
Mean (SD)	28.4 (4.53)
Median [Min, Max]	27.8 [15.7, 46.2]
Packyears [packs/day × years]	
Mean (SD)	3.46 (11.4)
Median [Min, Max]	0 [0, 133]
SES	
Low	99 (18.7%)
Medium	248 (46.8%)
High	183 (34.5%)
Location	
Rural	264 (49.8%)
Urban	266 (50.2%)
Season	
Winter	245 (46.2%)
Summer	285 (53.8%)
Diabetes	
No	455 (85.8%)
Yes	73 (13.8%)
Heating with fossil fuels	
No	478 (90.2%)
Yes	52 (9.8%)
Smoker	
No	513 (96.8%)
Yes	17 (3.2%)
Second smoker	
No	467 (88.1%)
Yes	63 (11.9%)
Regular sports	
Active	222 (41.9%)
Inactive	308 (58.1%)
Alcohol consumption	
Once a week or more often	135 (25.5%)
Less than once a week	395 (74.5%)

The description of temperature, air pollutants, and BP measurements is shown in Table 2. The daily average values were as follows: Tmean was 10.4 (± 7.56) °C, PM_2.5_ was 15.2 (± 9.04) µg/m^3^, NO_2_ was 23.5 (± 10.9) µg/m^3^, and O_3_ was 40.9 (± 20.5) µg/m^3^. While the means across the study sample of the daily averages for all air pollutants were below or very close to the 2021 World Health Organization (WHO) air quality guidelines (PM_2.5_ < 15 µg/m^3^, NO_2_ < 25 µg/m^3^), though some days exceeded these limits.

**Table 2:** Descriptive statistics of meteorological data, air pollution data, and blood pressure.

	Overall (*N* = 530)
Temperature in °C	
Mean (SD)	10.4 (7.57)
Median [Min, Max]	10.9 [− 4.52, 26.5]
Relative humidity in %	
Mean (SD)	77.9 (10.9)
Median [Min, Max]	80.4 [47.5, 95.9]
PM_2.5_ in µg/m^3^	
Mean (SD)	15.2 (9.06)
Median [Min, Max]	12.0 [3.90, 53.9]
NO_2_ in µg/m^3^	
Mean (SD)	23.5 (10.9)
Median [Min, Max]	23.4 [3.85, 55.3]
O_3_ in µg/m^3^	
Mean (SD)	41.0 (20.5)
Median [Min, Max]	41.5 [1.10, 86.3]
Systolic BP in mmHg	
Mean (SD)	145 (20.7)
Median [Min, Max]	144 [87.5, 248]
Diastolic BP in mmHg	
Mean (SD)	77.7 (10.1)
Median [Min, Max]	77.0 [50.0, 112]

The average SBP was generally observed to be high at around 145 (± 20.8) mmHg. However, the DBP was in the normal range (77.8 (± 10.1) mmHg Hg^20^).

Supplementary Figure S1 shows the individual relationships between BP and Tmean, as well as BP and air pollutants. We did not observe any general pattern among the exposures and BP. The estimated individual associations were mainly linear.

### The combined effect of temperature and air pollution on BP

**Fig. 1:**
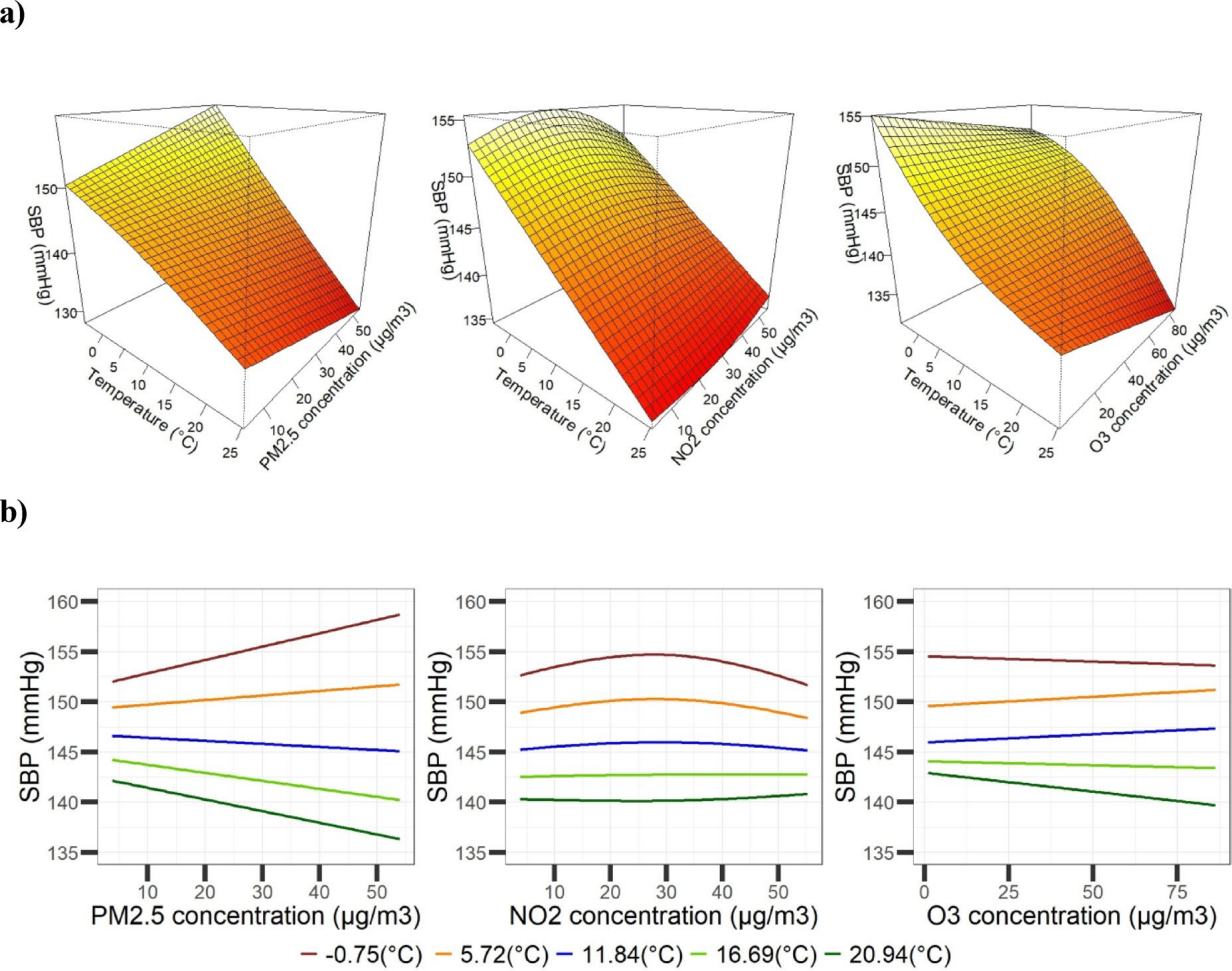
(*Panel* **a**) Visual representation of the bivariate response surfaces for SBP of temperature and air pollutants, PM_2.5_, NO_2_, and O_3_ (*column-wise*), estimated by the tensor product model. All models were adjusted for age, body mass index (BMI), socioeconomic status (SES), location (urban/rural), packyears, current smoking (yes/no), second smoker (yes/no), fossil fuel heating (yes/no), the season, and relative humidity (binary with cutoff 80%). The color intensity represents the magnitude of the BP values. Darker colors indicate lower values; lighter colors represent larger values. (*Panel* **b**) The adjusted associations between SBP and air pollutants at the 10th, 25th, 50th, 75th, and 90th percentiles of the Tmean distribution.

**Fig. 2:**
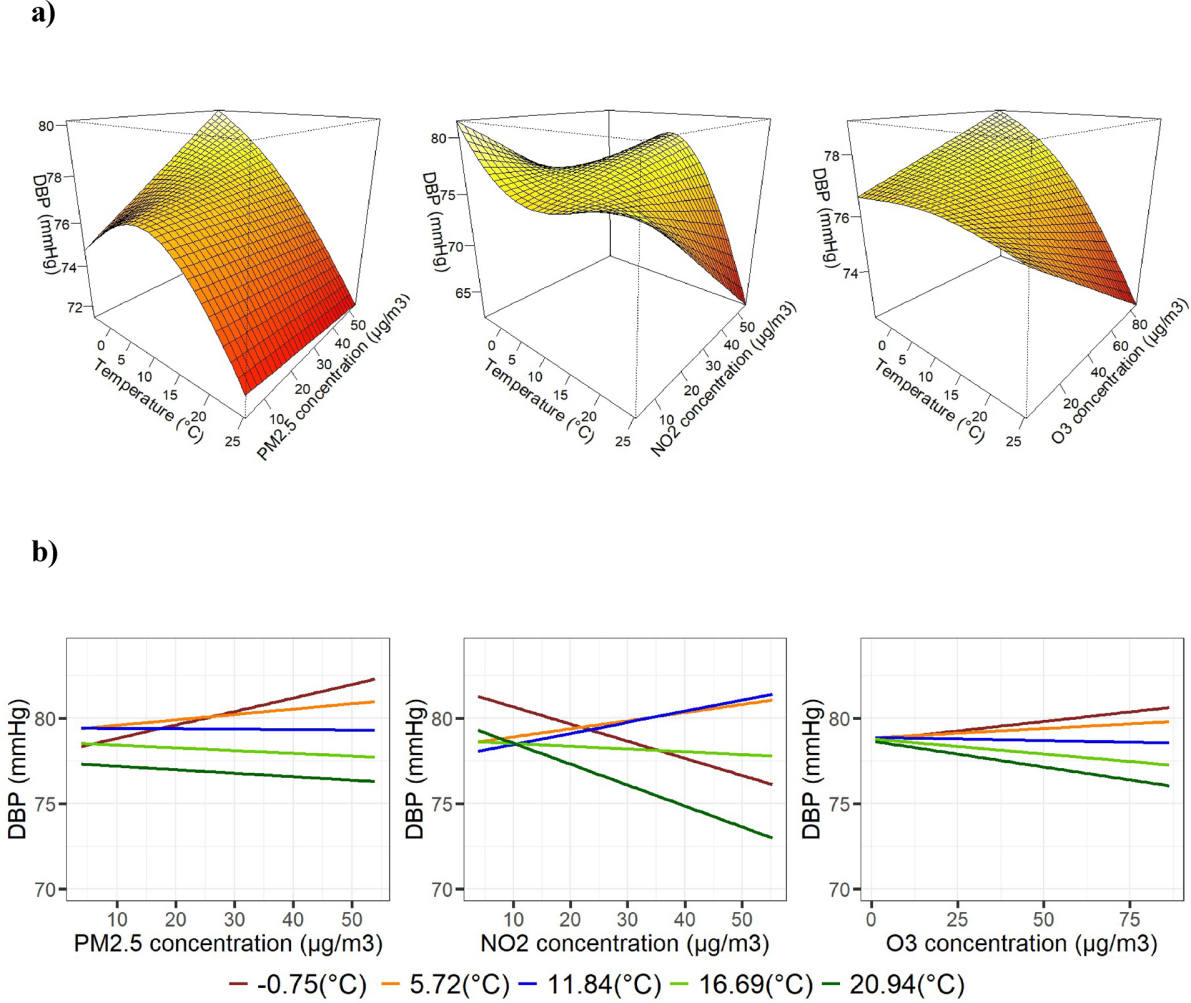
(*Panel* **a**) Visual representation of the bivariate response surfaces for DBP of temperature and air pollutants, PM_2.5_, NO_2_, and O_3_ (*column-wise*), estimated by the tensor product model. All models were adjusted for age, body mass index (BMI), socioeconomic status (SES), location (urban/rural), packyears, current smoking (yes/no), second smoker (yes/no), fossil fuel heating (yes/no), the season, and relative humidity. The color intensity represents the magnitude of the BP values. Darker colors indicate lower values; lighter colors represent larger values. (*Panel* **b**) The adjusted associations between DBP and air pollutants at the 10th, 25th, 50th, 75th, and 90th percentiles of the Tmean distribution.

Figures 1A and 2A display the 3D surface plots of SBP and DBP as functions of Tmean and air pollutants in the fitted model. These plots enable us to identify the relationships between Tmean and BP at any given concentration of air pollutants and between air pollutants and BP at every Tmean. Figures 1B and 2B show the associations between BP and air pollutants at the 10th, 25th, 50th, 75th, and 90th percentiles of the Tmean distribution. Overall, we observed a smooth, complex, non-linear combined effect of Tmean and air pollution on BP.

*The combined effect of temperature and PM*_*2.5*_* on BP*: At low temperatures, SBP and DBP increase with an increase in PM_2.5_ concentrations, whereas at high temperatures, SBP decreased as PM_2.5_ increased*.* The association between temperature and BP appeared approximately linear for SBP and non-linear for DBP (Figs. 1A and 2A). The percentile plots similarly indicate that higher PM_2.5_ concentrations were associated with higher SBP and DBP at lower temperatures (Figs. 1B and 2B).

*The combined effect of temperature and NO*_*2*_* on BP*: At lower temperatures, higher SBP was observed in association with increasing NO_2_ concentrations, whereas at higher temperatures, SBP was lower even at higher NO_2_ levels (Fig. 1A). Across all temperature categories, the association between NO_2_ and SBP showed a non-linear pattern. For DBP, a negative linear association with NO_2_ was observed at low and high temperatures, while at medium temperatures, DBP showed a positive association with increasing NO_2_ concentrations (Fig. 2A). These patterns are also reflected in the two-dimensional exposure–response surfaces (Figs. 1B and 2B).

*The combined effect of temperature and O*_*3*_* on BP*: Overall, SBP showed a negative linear association with both temperature and O_3_, such that lower SBP values were observed at higher temperature and O_3_ levels (Fig. 1A). A similar pattern was observed for DBP, although the association with temperature appeared slightly non-linear (Fig. 2A). Consequently, lower BP levels were observed across most temperature categories with increasing O_3_ concentrations, except at very low temperatures. Notably, the magnitude of this association varied across the temperature range, as illustrated by the percentile plots (Figs. 1B and 2B).

The smooth functions indicate largely linear age-related patterns, with lower SBP and modestly higher DBP observed with increasing age. Pack-years showed positive associations with both SBP and DBP, although uncertainty increased at higher exposure levels. These patterns were consistent across pollutant-specific models (Supplementary Figs. S2 and S3).

Across PM_2.5_, NO_2_, and O_3_ interaction models, BMI and SES were consistently inversely associated with SBP, whereas DBP showed consistent inverse associations with SES, RH, season, and smoking status, although none of these associations was statistically significant. Other covariates, including heating using fossil fuels and secondhand smoke exposure, were not significantly associated with either outcome. Effect estimates were similar across pollutant-specific models (Supplementary Tables S1 and S2). Supplementary Table S3 displays the significance of the bivariate tensor product on BP based on the *p*-values for the interaction between temperature and air pollution. We noticed that the combined effect of Tmean and air pollution was significantly associated with SBP, while the association with DBP was partly non-significant. Overall, the association between air pollution and BP differs with varying levels of Tmean, indicating an interactive effect of Tmean and air pollution on BP.

To assess potential effect modification by temperature, we compared the 90th and 10th percentiles of temperature. For SBP, comparing the 90th versus 10th percentile of temperature showed consistently negative differences across PM_2.5_, NO_2_, and O_3_, with confidence intervals that generally did not include zero, indicating statistically significant differences in pollutant effects between higher and lower temperatures (Supplementary Fig. S4). For DBP, most temperature categories had confidence intervals that included zero, suggesting limited statistical evidence of temperature-related effect modification (Supplementary Fig. S5). Notably, for NO_2_, both lower and higher temperature percentiles showed similar inverse associations with DBP, indicating little difference in effect across temperature levels (Fig. 2B).

### Seasonal effect modification of temperature-pollutant associations

**Fig. 3:**
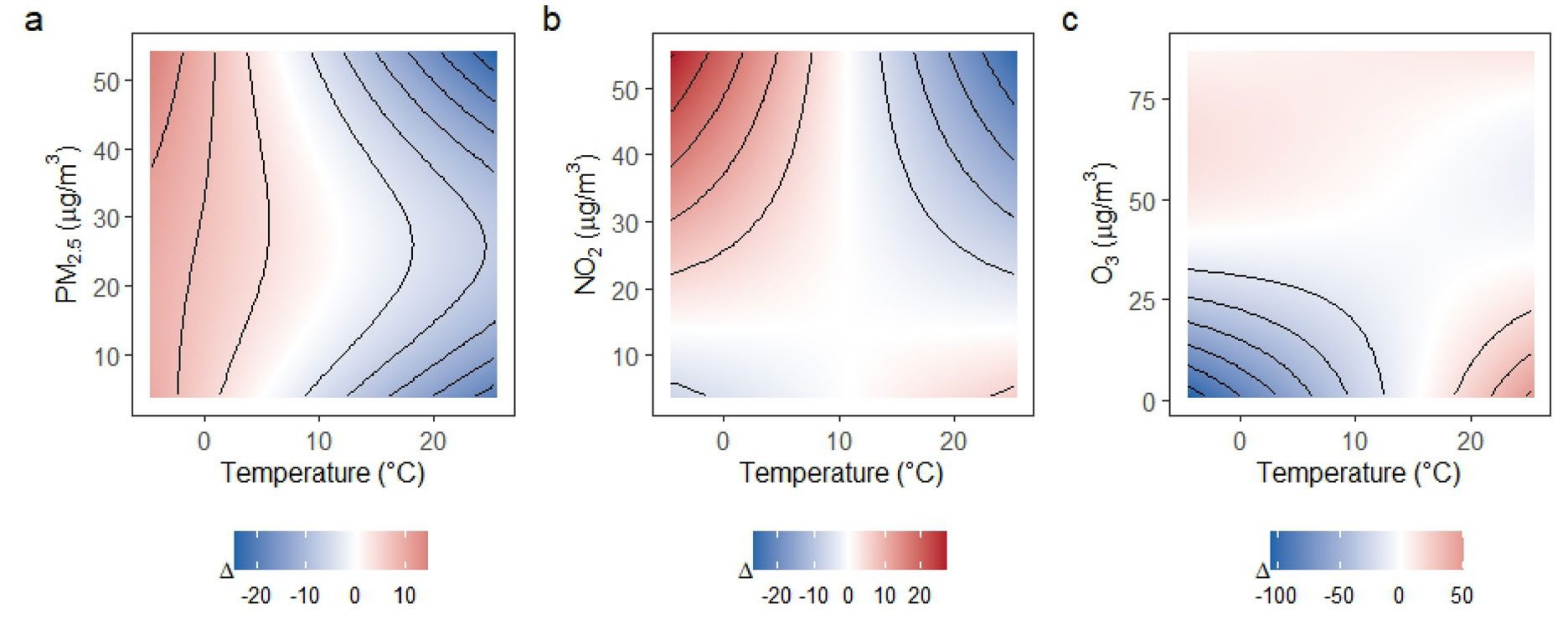
Seasonal difference surfaces for the joint associations of temperature and air pollutants with SBP. Panels (**a**–**c**) display the estimated difference in the fitted SBP response between the warm season (April–September) and the cold season (October–March) for PM_2.5_, NO_2_, and O_3_, respectively. Colors represent the magnitude and direction of the seasonal difference (warm minus cold season), with red indicating stronger associations in the warm season and blue indicating stronger associations in the cold season. Contour lines depict smooth gradients of the difference surface. Black dots indicate regions where the seasonal difference is statistically significant on a pointwise basis. All other covariates were held constant at typical values.

**Fig. 4:**
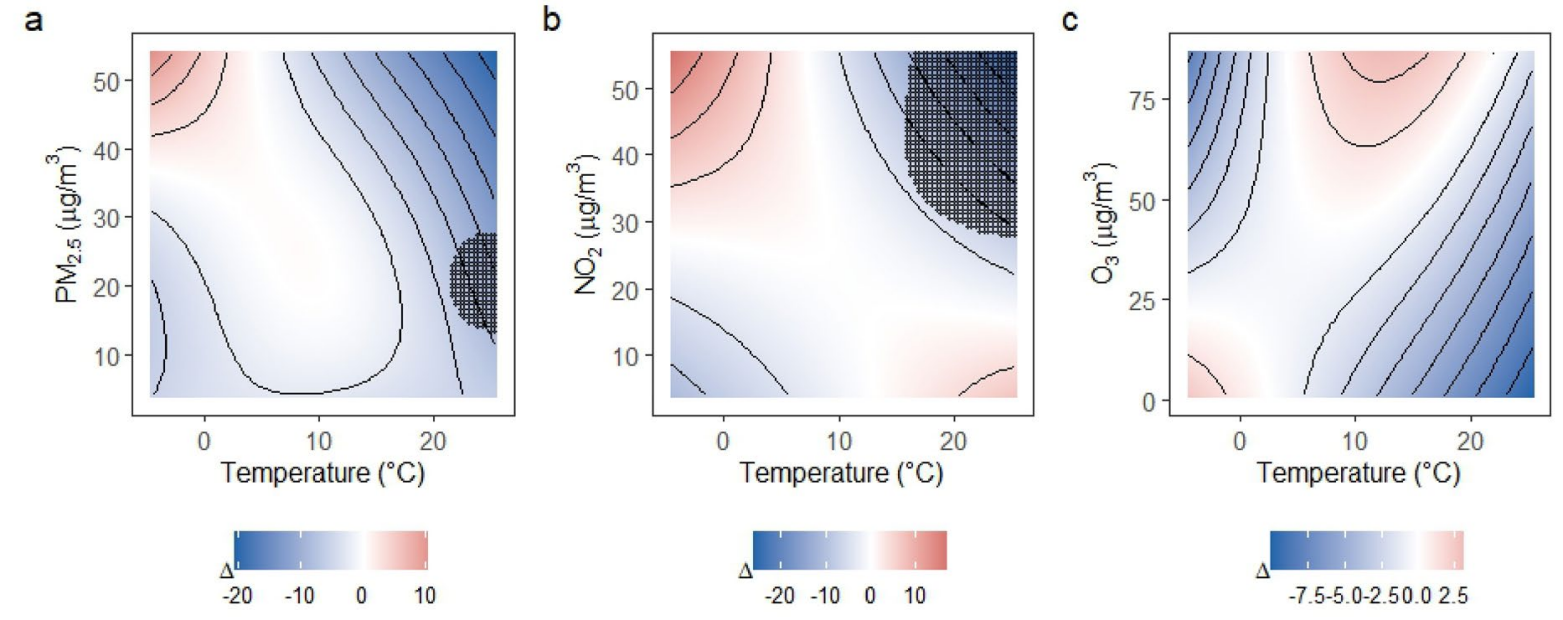
Seasonal difference surfaces for the joint associations of temperature and air pollutants with DBP. Panels (**a**–**c**) display the estimated difference in the fitted DBP response between the warm season (April–September) and the cold season (October–March) for PM_2.5_, NO_2_, and O_3_, respectively. Colors represent the magnitude and direction of the seasonal difference (warm minus cold season), with red indicating stronger associations in the warm season and blue indicating stronger associations in the cold season. Contour lines depict smooth gradients of the difference surface. Black dots indicate regions where the seasonal difference is statistically significant on a pointwise basis. All other covariates were held constant at typical values.

The difference surfaces, representing the model-estimated SBP contrast between warm and cold seasons across pollutant concentrations, revealed clear and pollutant-specific seasonal patterns. For SBP, the joint effects of PM_2.5_ and NO_2_ were generally stronger during the cold season, especially at higher pollutant concentrations and at moderate to high temperatures. In contrast, O_3_ exhibited the opposite pattern, with stronger associations predominantly during the warm season, particularly at elevated temperatures (Fig. 3). For DBP, seasonal modification was weaker and more localized (Fig. 4). Significant seasonal differences were confined to limited regions of the exposure domain, primarily at higher concentrations of PM_2.5_ and NO_2_, whereas O_3_ showed little to no robust seasonal modification. Across pollutants, seasonal modification of DBP was generally localized and often absent, especially when compared with SBP.

### Stratification analysis

We studied the combined effect of Tmean and air pollution on BP, stratified by location, SES, and BMI (Supplementary Tables S4, S5, and S6). A BMI above 25 kg/m^2^ is classified as high according to WHO guidelines. Overall, we found significant combined effects of Tmean and air pollutants on both SBP and DBP in women living in urban areas and in those with low to medium SES. However, no significant combined effect was observed among older individuals with high BMI.

### Sensitivity analysis

We performed several sensitivity analyses to assess the robustness of the results. Additional adjustment for alcohol intake frequency, physical activity, and diabetes did not substantially change the findings of the core model. We also assessed the degree of non-linearity in the bivariate exposure–response surface by estimating the effective degrees of freedom (edf) values using both GCV scores and the REML method in the GAM model. Larger edf values indicate greater non-linearity, while an edf of 1 signifies a linear surface. The edf for the combined effect of Tmean and PM_2.5_ concentration was estimated at 4.02 (REML) and 4.18 (GCV), respectively.

Delayed exposure analyses for lag 0–1 and lag 0–3 are presented in the Supplementary Material. Supplementary Figs. S6 and S7 show the estimated delayed associations with SBP for lag 0–1 and lag 0–3, respectively. Overall, the combined exposure response patterns for PM_2.5_ and NO_2_ were similar to those observed at lag0 (same day), with a positive, approximately linear association between NO_2_ and SBP across the temperature range. For DBP, the PM_2.5_ association became more non-linear with increasing lag window, whereas the patterns for NO_2_ and O_3_ were broadly comparable to the lag0 results (Supplementary Figs. S8 and S9).

## Discussion

Our study within the SALIA cohort examines the non-linear relationships between temperature, air pollution, and BP. We observed that the combined association of temperature and air pollution with BP remained pronounced among older adults after adjustment for individual characteristics, season, and RH. To our knowledge, this is the first study to use a bivariate non-linear exposure–response surface to model the combined impact of air pollutants and temperature on BP. Specifically, exposure to low temperatures combined with higher concentrations of pollutants such as PM_2.5_ and NO_2_ was associated with increased BP.

Previous studies have examined the short-term effects of temperature and air pollution on BP, often modelling these relationships as linear. Moreover, the evidence coming from these studies is inconsistent. Some studies report positive associations between temperature/air pollution and BP^2,21,22^, while others report negative associations^23,24^. Most of these studies, however, examined the individual effects of temperature and pollution, while the evidence of their combined effect is scarce. Among the existing studies that modeled the combined effect of temperature, air pollution and BP, most studies employed linear interaction terms to model the combined effect, suggesting that low temperatures and high pollution may elevate BP in healthy adults^25^. However, such approaches are limited in capturing non-linear relationships and often yield non-significant results. More recent studies examining short-^26^ and long-term^27^ exposures have identified non-linear associations between certain pollutants (O_3_, NO_2_) and SBP, but not with PM. The present study, on the other hand, offers a more flexible framework to examine the effects of PM_2.5_, NO_2_, and O_3_ exposures on BP across varying temperature levels, while allowing for both linear and non-linear relationships. Consistent with the previous studies, our study also indicated a linear combined effect of temperature and PM_2.5_ on SBP, while the combined effect with NO_2_ was non-linear with BP.

We observed higher BP levels associated with PM_2.5_ exposure at lower temperature ranges, whereas lower BP levels were observed at moderate to higher temperatures. While most previous studies have reported positive associations between PM_2.5_ and BP^21,28^, and some report no significant association^29^, our findings suggest that this relationship varies with temperature, indicating temperature-dependent heterogeneity. The relationship between BP and other air pollutants is also quite inconsistent in previous studies. A Danish cohort study and others have reported lower SBP levels associated with increasing NO_2_ exposure^30,31^. In our analysis, SBP showed a non-linear association, while DBP showed lower values at low to high temperatures with increasing NO_2_, but higher values at moderate temperatures. Despite observing a generally negative association between O_3_ and BP, our findings contrast with another panel study reporting increased DBP following a short-term increase in O_3_ exposure^32^. Nevertheless, a recent narrative review highlights heterogeneous short-term BP responses to O_3_, including non-significant and inverse associations, potentially driven by competing vascular and cardiac compensatory mechanisms^33^. These inconsistencies may be explained by differences in study populations, geographic regions, exposure assessment, sample size, and the extent to which temperature-related effect modification was considered. Our findings further suggest that failure to account for the non-linear association between temperature, air pollution, and BP may contribute to these discrepancies.

We found that the association between temperature, air pollution, and BP was stronger among women living in urban areas compared to those in rural areas. This is consistent with previous findings showing higher BP following relocation from suburban to metropolitan environments, potentially due to increased exposure to air pollution and other urban stressors such as traffic-related noise^34^. In addition, stronger associations observed in lower SES groups align with prior studies linking air pollution to hypertension among women with lower income and educational attainment^35^.

We observed clear seasonal modification of the joint temperature-air pollution association with BP, with pollutant-specific patterns. PM_2.5_ and NO_2_ showed stronger associations during the cold season, consistent with higher combustion-related emissions and altered exposure conditions in colder months. In contrast, O_3_ effects were stronger in the warm season, reflecting its dependence on high temperature and bright sunshine. Seasonal modification was more pronounced for SBP than for DBP, suggesting greater sensitivity of SBP to season-dependent vascular and autonomic responses. These findings underscore the importance of accounting for seasonal context when evaluating the effect of environmental exposures on cardiovascular diseases.

The biological mechanisms linking air pollution and BP are not yet fully understood. One widely accepted theory suggests that PM induces oxidative reactions and systemic inflammation, leading to vascular dysfunction^36^. Aging arteries have reduced antioxidant capacity, resulting in heightened oxidative stress. This compromises endothelial and vasomotor functions, impairing BP autoregulation during inflammation^37^. This may explain why older adults experience greater BP increases from air pollution than younger individuals^38^. In addition, ultrafine particles may penetrate the alveolar barrier and directly affect endothelial function^39^. The relationship between temperature and BP is more established. Cold exposure activates the sympathetic nervous system, increasing heart rate and BP, whereas higher temperatures may reduce BP (hypotension) through decreased peripheral resistance and increased blood viscosity (due to dehydration)^7^.

Our study has several strengths. Firstly, the use of a two-dimensional exposure–response surface allows flexible modeling of the joint effects of temperature and air pollution, accommodating different measurement scales and capturing non-linear relationships. Compared with linear interaction models, this approach reduces power loss associated with stratification and provides a more nuanced representation of complex exposure patterns^40^. Secondly, our well-characterized cohort, including detailed information on individual-level characteristics and lifestyle factors such as smoking and alcohol consumption, provides a solid basis for controlling potential confounding.

We also acknowledge several limitations of our study. The study population consisted of older women from the industrialized area of Germany. Thus, the conclusions may not be generalized to other study populations. The cross-sectional nature of the study restricts causal interpretation since exposure and outcome are measured simultaneously. Residual confounding cannot be ruled out, particularly due to the lack of detailed information on antihypertensive medication use.

Additional factors may also influence our findings. Retirement status, which may affect daily activity patterns and exposure profiles, was not available and could not be accounted for in the analysis. While we adjusted for long-term physical activity, short-term activity prior to BP measurement could not be considered. Finally, information on hormone replacement therapy (HRT) and antihypertensive medication use was available but not included in the current analysis. Evidence linking HRT to short-term BP fluctuations is limited and inconsistent, and its potential to modify the associations between temperature, air pollution, and BP remains unclear. Moreover, medication use was not categorized into specific classes, precluding adjustment for antihypertensive treatment, which may influence measured BP levels. These limitations should be considered when interpreting the results.

## Conclusion

In conclusion, we observed an increase in SBP and DBP in older German women exposed to low temperatures and high levels of air pollution. Unlike most previous studies, which have used the linear interaction model to analyze the combined effect of temperature and air pollution, we propose using non-linear bivariate tensor splines. Future studies should explore the combined effects of temperature and air pollution on BP and investigate biological pathways to clarify these complex interactions. The findings of this research highlight the urgent need to address the intricate synergy between environmental variables and public health outcomes.

## Supplementary Information

Below is the link to the electronic supplementary material.

Supplementary Material 1

## Data Availability

The datasets used and/or analyzed during the current study available from the corresponding author on reasonable request.

## References

[1] World Health Organization. Air pollution—who.int. https://www.who.int/news-room/fact-sheets/detail/ambient-(outdoor)-air-quality-and-health (2022). Accessed 8 Feb. 2023.

[2] de Bont, J. et al. Ambient air pollution and cardiovascular diseases: An umbrella review of systematic reviews and meta-analyses. *J. Intern. Med.* **291**, 779–800 (2022). 35138681 10.1111/joim.13467 PMC9310863

[3] Yang, B. Y. et al. Global association between ambient air pollution and blood pressure: A systematic review and meta-analysis. *Environ. Pollut.* **235**, 576–588 (2018). 29331891 10.1016/j.envpol.2018.01.001

[4] Nemmar, A., Subramaniyan, D., Yasin, J. & Ali, B. H. Impact of experimental type 1 diabetes mellitus on systemic and coagulation vulnerability in mice acutely exposed to diesel exhaust particles. *Part. Fibre Toxicol.* **10**, 1–10 (2013). 23587270 10.1186/1743-8977-10-14 PMC3641025

[5] Wilson, S. J., Miller, M. R. & Newby, D. E. Effects of diesel exhaust on cardiovascular function and oxidative stress. *Antioxid. Redox Signal.* **28**, 819–836 (2018). 28540736 10.1089/ars.2017.7174

[6] Modesti, P. A. Season, temperature and blood pressure: A complex interaction. *Eur. J. Intern. Med.* **24**, 604–607 (2013). 23972926 10.1016/j.ejim.2013.08.002

[7] Wang, Q. et al. Environmental ambient temperature and blood pressure in adults: A systematic review and meta-analysis. *Sci. Total Environ.* **575**, 276–286 (2017). 27750133 10.1016/j.scitotenv.2016.10.019

[8] Analitis, A. et al. Synergistic effects of ambient temperature and air pollution on health in Europe: Results from the PHASE project. *Int. J. Environ. Res. Public Health.* **15**, 1856 (2018). 30154318 10.3390/ijerph15091856 PMC6163671

[9] Mokoena, K. K. et al. Interaction effects of air pollution and climatic factors on circulatory and respiratory mortality in Xi’an, China between 2014 and 2016. *Int. J. Environ. Res. Public Health.* **17**, 9027 (2020). 33287400 10.3390/ijerph17239027 PMC7729743

[10] Zhou, L. et al. The interactive effects of extreme temperatures and PM2.5 pollution on mortalities in Jiangsu Province, China. *Sci. Rep.* **13**, 9479 (2023). 37301905 10.1038/s41598-023-36635-x PMC10257702

[11] De Vita, A. et al. The impact of climate change and extreme weather conditions on cardiovascular health and acute cardiovascular diseases. *J. Clin. Med.* **13**, 759 (2024). 38337453 10.3390/jcm13030759 PMC10856578

[12] Areal, A. T., Zhao, Q., Wigmann, C., Schneider, A. & Schikowski, T. The effect of air pollution when modified by temperature on respiratory health outcomes: A systematic review and meta-analysis. *Sci. Total Environ.* **811**, 152336 (2022). 34914983 10.1016/j.scitotenv.2021.152336

[13] Simoni, M. et al. Adverse effects of outdoor pollution in the elderly. *J. Thorac. Dis.* **7**, 34 (2015). 25694816 10.3978/j.issn.2072-1439.2014.12.10 PMC4311079

[14] Wood, S. N. *Generalized Additive Models: An Introduction with R* (Chapman and Hall/CRC, 2017).

[15] Schikowski, T. et al. Long-term air pollution exposure and living close to busy roads are associated with COPD in women. *Respir. Res.* **6**, 1–10 (2005). 16372913 10.1186/1465-9921-6-152 PMC1352358

[16] Ohlwein, S. et al. Air pollution and diastolic function in elderly women—Results from the SALIA study cohort. *Int. J. Hyg. Environ. Health* **219**, 356–363 (2016). 27009693 10.1016/j.ijheh.2016.02.006

[17] Bollmeyer, C. et al. Towards a high-resolution regional reanalysis for the European CORDEX domain. *Q. J. R. Meteorol. Soc.* **141**, 1–5 (2015).

[18] Minkos, A., Dauert, U., Feigenspan, S. & Kessenger, S. *Air Quality 2016: Preliminary Evaluation* (Umweltbundesamt, 2017).

[19] R Core Team. R: A Language and Environment for Statistical Computing. *R Foundation for Statistical Computing.* https://www.R-project.org/ (2024).

[20] Chobanian, A. V. et al. Seventh report of the joint national committee on prevention, detection, evaluation, and treatment of high blood pressure. *Hypertension* **42**, 1206–1252 (2003). 14656957 10.1161/01.HYP.0000107251.49515.c2

[21] Ishii, M. et al. Association of short-term exposure to Asian dust with increased blood pressure. *Sci. Rep.* **10**, 17630 (2020). 33077773 10.1038/s41598-020-74713-6 PMC7572380

[22] Wen, T. et al. Short-term air pollution levels and blood pressure in older women. *Epidemiology* **34**, 271–281 (2023). 36722810 10.1097/EDE.0000000000001577 PMC9891284

[23] Chen, Q. et al. Association between ambient temperature and blood pressure and blood pressure regulators: 1831 hypertensive patients followed up for three years. *PLoS ONE* **8**, e84522 (2013). 24391962 10.1371/journal.pone.0084522 PMC3877276

[24] Giorgini, P. et al. Particulate matter air pollution and ambient temperature: Opposing effects on blood pressure in high-risk cardiac patients. *J. Hypertens.* **33**, 2032–2038 (2015). 26203968 10.1097/HJH.0000000000000663

[25] Wu, S. et al. Does ambient temperature interact with air pollution to alter blood pressure? A repeated-measure study in healthy adults. *J. Hypertens.* **33**, 2414–2421 (2015). 26378686 10.1097/HJH.0000000000000738

[26] Choi, Y. J. et al. Short-term effects of air pollution on blood pressure. *Sci. Rep.* **9**, 20298 (2019). 31889065 10.1038/s41598-019-56413-y PMC6937254

[27] Arku, R. E. et al. Long-term exposure to outdoor and household air pollution and blood pressure in the prospective urban and rural epidemiological (PURE) study. *Environ. Pollut.* **262**, 114197 (2020). 32146361 10.1016/j.envpol.2020.114197 PMC7767575

[28] Brook, R. D. et al. Differences in blood pressure and vascular responses associated with ambient fine particulate matter exposures measured at the personal versus community level. *Occup. Environ. Med.* **68**, 224–230 (2011). 20935292 10.1136/oem.2009.053991

[29] Lee, D. H. et al. Personal exposure to fine particulate air pollutants impacts blood pressure and heart rate variability. *Sci. Rep.* **10**, 16538 (2020). 33024194 10.1038/s41598-020-73205-x PMC7538889

[30] Sørensen, M. et al. Long-term exposure to traffic-related air pollution associated with blood pressure and self-reported hypertension in a Danish cohort. *Environ. Health Perspect.* **120**, 418–424 (2012). 22214647 10.1289/ehp.1103631 PMC3295339

[31] Floyd, C. N. et al. Acute blood pressure-lowering effects of nitrogen dioxide exposure from domestic gas cooking via elevation of plasma nitrite concentration in healthy individuals. *Circ. Res.* **127**, 847–848 (2020). 32539547 10.1161/CIRCRESAHA.120.316748 PMC7447162

[32] Song, J. et al. Short-time exposure to ambient ozone and associated cardiovascular effects: A panel study of healthy young adults. *Environ. Int.* **137**, 105579 (2020). 32086080 10.1016/j.envint.2020.105579

[33] Hua, Q. et al. Ozone exposure and cardiovascular disease: A narrative review of epidemiology evidence and underlying mechanisms. *Fundam. Res.* **5**(1), 249–263 (2025). 40166088 10.1016/j.fmre.2024.02.016 PMC11955045

[34] Wu, S. et al. Blood pressure changes and chemical constituents of particulate air pollution: Results from the healthy volunteer natural relocation (HVNR) study. *Environ. Health Perspect.* **121**, 66–74 (2013). 23086577 10.1289/ehp.1104812 PMC3546346

[35] Abba, M. S. et al. Household air pollution and high blood pressure: A secondary analysis of the 2016 Albania Demographic Health and Survey dataset. *Int. J. Environ. Res. Public Health* **19**, 2611 (2022). 35270304 10.3390/ijerph19052611 PMC8909881

[36] Arias-Pérez, R. D. et al. Inflammatory effects of particulate matter air pollution. *Environ. Sci. Pollut. Res.* **27**, 42390–42404 (2020). 10.1007/s11356-020-10574-w 32870429

[37] Zhou, X., Bohlen, H. G., Unthank, J. L. & Miller, S. J. Abnormal nitric oxide production in aged rat mesenteric arteries is mediated by NAD(P)H oxidase-derived peroxide. *Am. J. Physiol. Heart Circ. Physiol.* **297**, H2227–H2233 (2009). 19783779 10.1152/ajpheart.00325.2009 PMC2793129

[38] Nicolaou, L. et al. Cross-sectional analysis of the association between personal exposure to household air pollution and blood pressure in adult women: Evidence from the multi-country Household Air Pollution Intervention Network (HAPIN) trial.. *Environ. Res.* **214**, 114121 (2022). 36029836 10.1016/j.envres.2022.114121 PMC9492861

[39] Schraufnagel, D. E. The health effects of ultrafine particles.. *Exp. Mol. Med.* **52**, 311–317 (2020). 32203102 10.1038/s12276-020-0403-3 PMC7156741

[40] Royston, P., Altman, D. G. & Sauerbrei, W. Dichotomizing continuous predictors in multiple regression: A bad idea.. *Stat. Med.* **25**, 127–141 (2006). 16217841 10.1002/sim.2331

