# Supplementary material for: Synergistic effects of air pollution and temperature on blood pressure in older German women
Source: Sci Rep. 2026 May 13;16:15046. doi: 10.1038/s41598-026-51334-z (PMC13172101; doi:10.1038/s41598-026-51334-z)
Supplement: Supplementary file 1 — Supplementary Material 1 [file 41598_2026_51334_MOESM1_ESM.docx]

**Supplementary Material: Synergistic Effects of Air Pollution and Temperature on Blood Pressure in Older German Women**

1. Dayasri Ravia, Andreas Grolla, Claudia Wigmannb, Nidhi Singhb, Tamara Schikowskib,c
2. *aDepartment of Statistics, TU Dortmund University, Vogelpothsweg 87,*
3. *Dortmund, 44227, NRW, Germany*
4. *bIUF-Leibniz Research Institute for Environmental Medicine, Auf ’m Hennekamp 50, Düsseldorf, 40225, NRW, Germany*
5. *cSchool of Public Health, Department of Environment and Health, University of Bielefeld,*
   - 1. *33501 Bielefeld, Germany*

Table S1: Estimated parametric coefficients (β) and 95% confidence intervals from generalized additive models assessing the association between temperature–pollutant interactions and SBP.

|  | PM_2.5_ (µg/m^3^) | NO_2_ (µg/m^3^) | O_3_ (µg/m^3^) |
| --- | --- | --- | --- |
| BMI in kg/m^2^ | -0.207 (-0.626,0.213) | -0.215 (-0.635,0.204) | -0.209 (-0.629,0.211) |
| SES | -2.304 (-5.024,0.416) | -2.404 (-5.133,0.325) | -2.359 (-5.085,0.366) |
| Heating with fossil fuels | 1.709 (-4.962,8.379) | 1.585 (-5.110,8.281) | 1.621 (-5.065,8.307) |
| Location | 3.182 (-1.511,7.875) | 2.839 (-2.275,7.953) | 2.927 (-1.585,7.439) |
| Relative humidity in % | 0.501 (-3.764,4.765) | 0.593 (-3.737,4.923) | 0.324 (-4.384,5.032) |
| Season | -0.382 (-6.413,5.649) | 0.260 (-6.162,6.682) | -0.312 (-6.770,6.145) |
| Second smoke | -0.185 (-6.299,5.930) | -0.363 (-6.483,5.757) | -0.316 (-6.438,5.805) |
| Smoker | 2.172 (-11.070,15.414) | 2.315 (-10.953,15.582) | 2.177 (-11.068,15.422) |

Table S2: Estimated parametric coefficients (β) and 95% confidence intervals from generalized additive models assessing the association between temperature–pollutant interactions and DBP.

|  | PM_2.5_ (µg/m^3^) | NO_2_ (µg/m^3^) | O_3_ (µg/m^3^) |
| --- | --- | --- | --- |
| BMI in kg/m^2^ | 0.034 (-0.174,0.241) | 0.025 (-0.181,0.230) | 0.028 (-0.179,0.236) |
| SES | -0.477 (-1.819,0.865) | -0.455 (-1.792,0.883) | -0.463 (-1.808,0.883) |
| Heating with fossil fuels | 1.456 (-1.833,4.745) | 1.485 (-1.790,4.761) | 1.309 (-1.993,4.611) |
| Location | 1.000 (-1.325,3.325) | 1.434 (-1.081,3.948) | 0.777 (-1.448,3.002) |
| Relative humidity in % | -0.642 (-2.817,1.533) | -0.233 (-2.465,1.999) | -0.273 (-2.592,2.046) |
| Season | -1.140 (-4.135,1.855) | -0.910 (-4.101,2.282) | -0.939 (-4.093,2.215) |
| Second smoke | 1.155 (-1.860,4.170) | 1.259 (-1.743,4.261) | 1.132 (-1.890,4.154) |
| Smoker | -1.186 (-7.716,5.344) | -0.297 (-6.827,6.232) | -1.078 (-7.613,5.458) |

|  |
| --- |
|  |

Table S3: *p*-values of the bivariate tensor product between air pollution and temperature (*p*-values<0.05 in bold).

|  | PM_2.5_ | NO_2_ | O_3_ |
| --- | --- | --- | --- |
| SBP | 0.03 | 0.04 | 0.03 |
| DBP | 0.23 | **0.04** | 0.27 |

Table S4: Statistical significance of the combined effect between air pollution and temperature in different locations (*p*-values<0.05 in bold).

| Location | BP | PM_2.5_ | NO_2_ | O_3_ |
| --- | --- | --- | --- | --- |
| Rural | SBP | 0.32 | 0.47 | 0.22 |
| (N=271) | DBP | 0.67 | 0.59 | 0.98 |
| Urban | SBP | **0.03** | **0.04** | **0.01** |
| (N=270) | DBP | **0.02** | **0.03** | **0.02** |

Table S5: Statistical significance of the combined effect between air pollution and temperature in different SES status (*p*-values<0.05 in bold).

| SES | BP | PM_2.5_ | NO_2_ | O_3_ |
| --- | --- | --- | --- | --- |
| Low | SBP | **0.01** | **0.00** | **0.00** |
| (N=101) | DBP | 0.13 | 0.16 | 0.06 |
| Medium | SBP | 0.08 | **0.00** | **0.01** |
| (N=251) | DBP | 0.09 | **0.02** | **0.04** |
| High | SBP | 0.28 | 0.78 | 0.85 |
| (N=189) | DBP | 0.06 | 0.72 | 0.93 |

Table S6: Statistical significance of the combined effect between air pollution and temperature in different groups of BMI (*p*-values<0.05 in bold).

| BMI | BP | PM_2.5_ | NO_2_ | O_3_ |
| --- | --- | --- | --- | --- |
| Low BMI | SBP | 0.13 | 0.16 | 0.19 |
| (N=111) | DBP | 0.65 | 0.77 | 0.75 |
| High BMI | SBP | 0.15 | 0.14 | 0.18 |
| (N=430) | DBP | 0.17 | **0.02** | 0.07 |


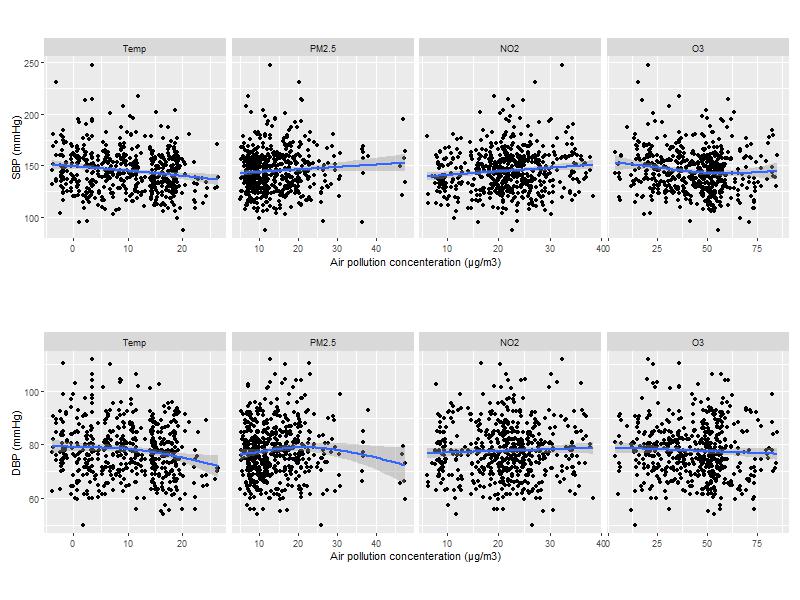
Figure S1: Individual relationships between Tmean (*◦*C) / air pollution concentrations (µg*/*m3) and SBP (mmHg) / DBP (mmHg). The blue curve in the plot represents the modeled trend derived from a Generalized Additive Model (GAM) fit. For illustration, a simple GAM model with exposures and BP is used. *Shadows* represent the 95% confidence intervals (CIs).


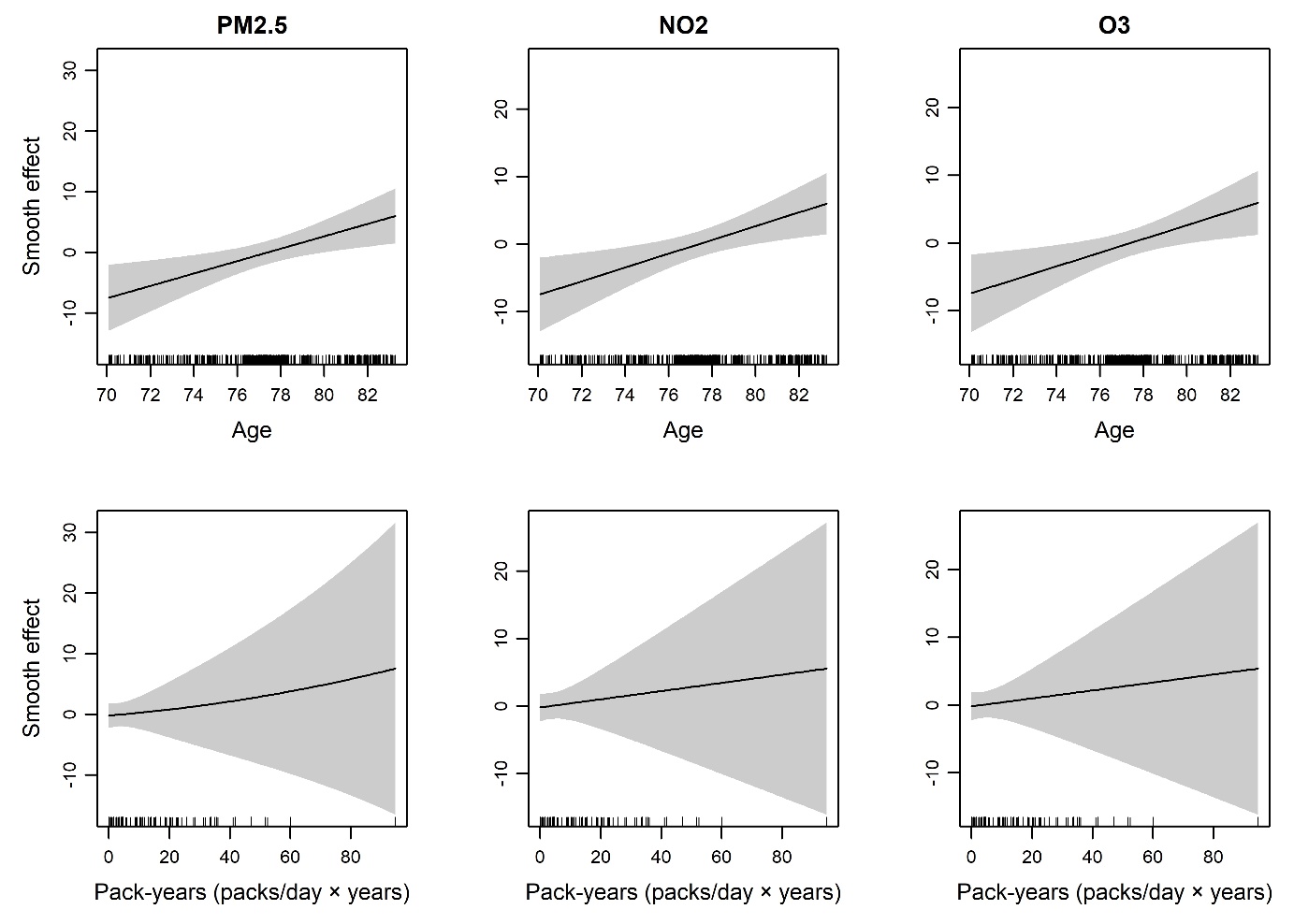


Figure S2: Smooth functions of age (top row) and cumulative smoking exposure (pack-years; bottom row) from generalized additive models of SBP for PM_2.5_, NO_2_, and O_3_ models. Shaded areas represent 95% confidence intervals.


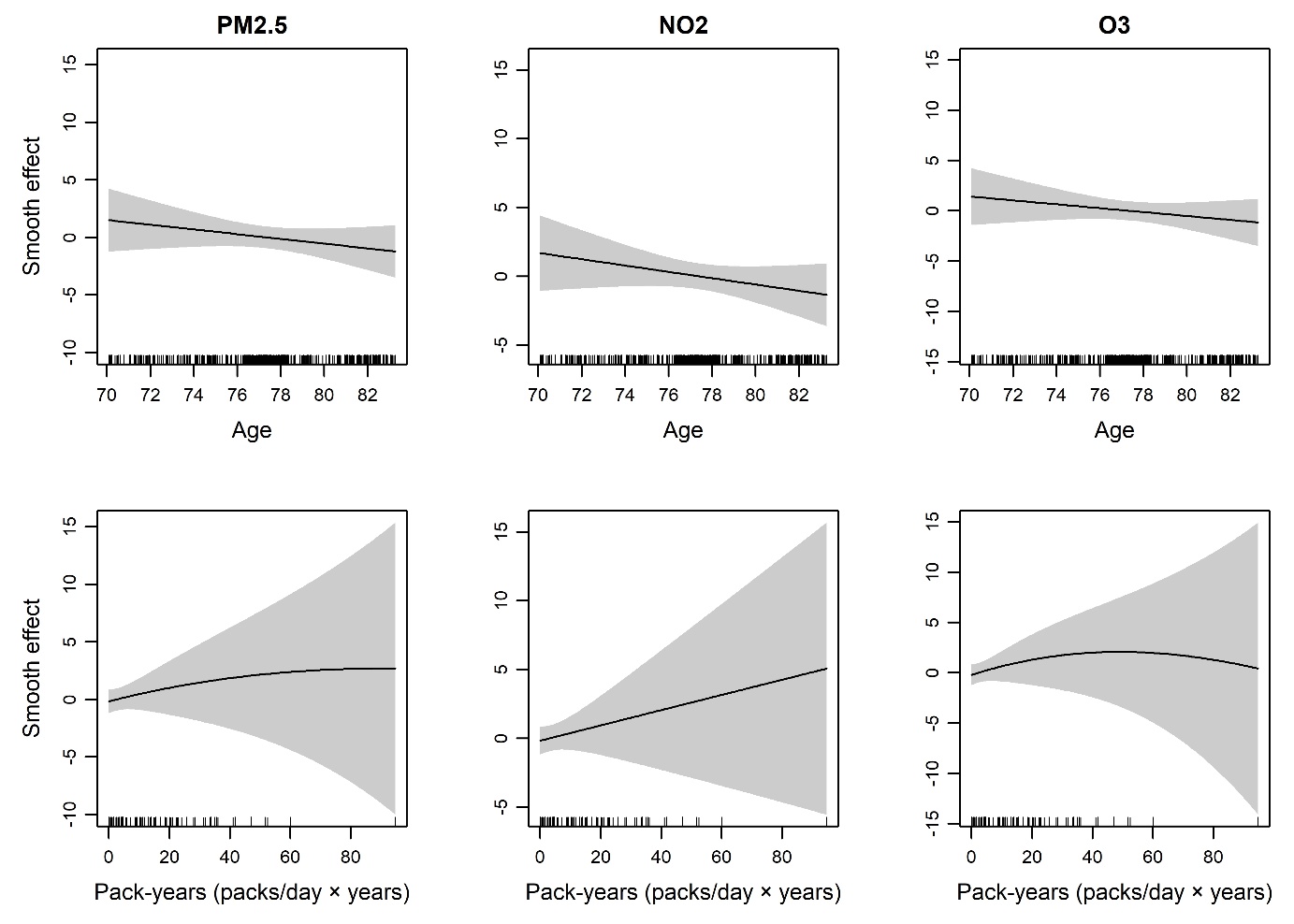


Figure S3: Smooth functions of age (top row) and cumulative smoking exposure (pack-years; bottom row) from generalized additive models of DBP for PM_2.5_, NO_2_, and O_3_ models. Shaded areas represent 95% confidence intervals.


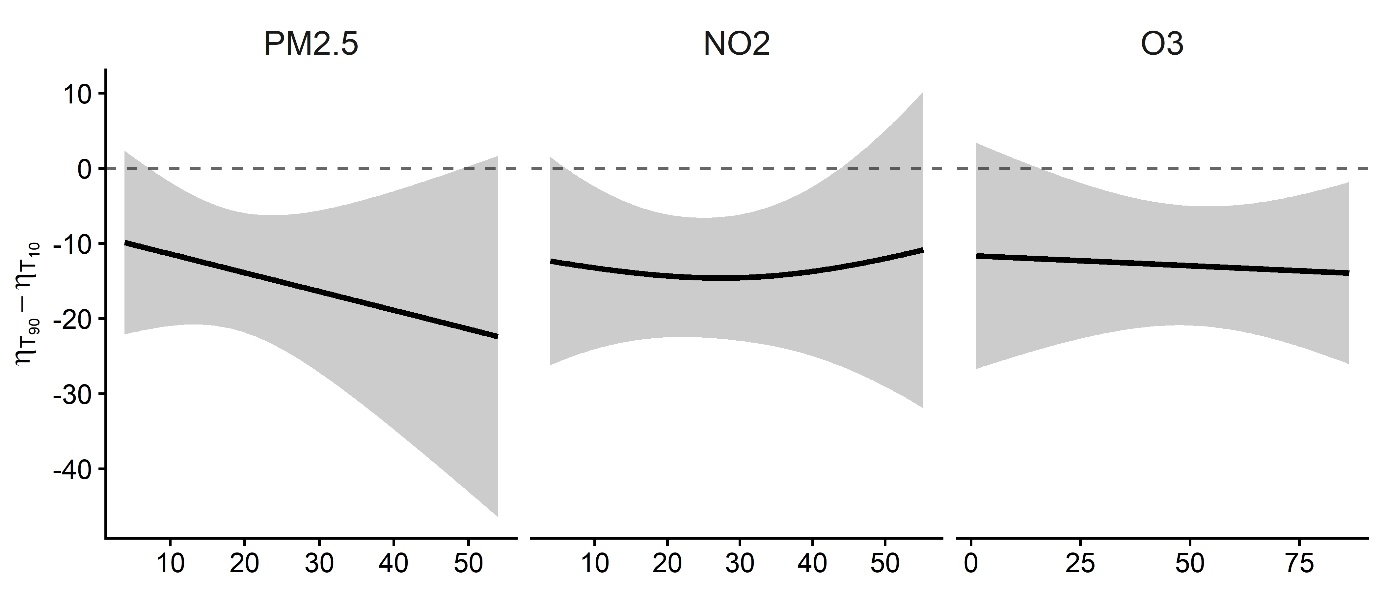


Figure S4: Estimated difference in the linear predictor comparing the 90^th^ versus 10^th^ percentile of temperature (η_{T90} − η_{T10}) across pollutant concentrations from generalized additive models of SBP. Curves are shown separately for PM_2.5_, NO_2_, and O₃. Shaded areas represent 95% confidence intervals.


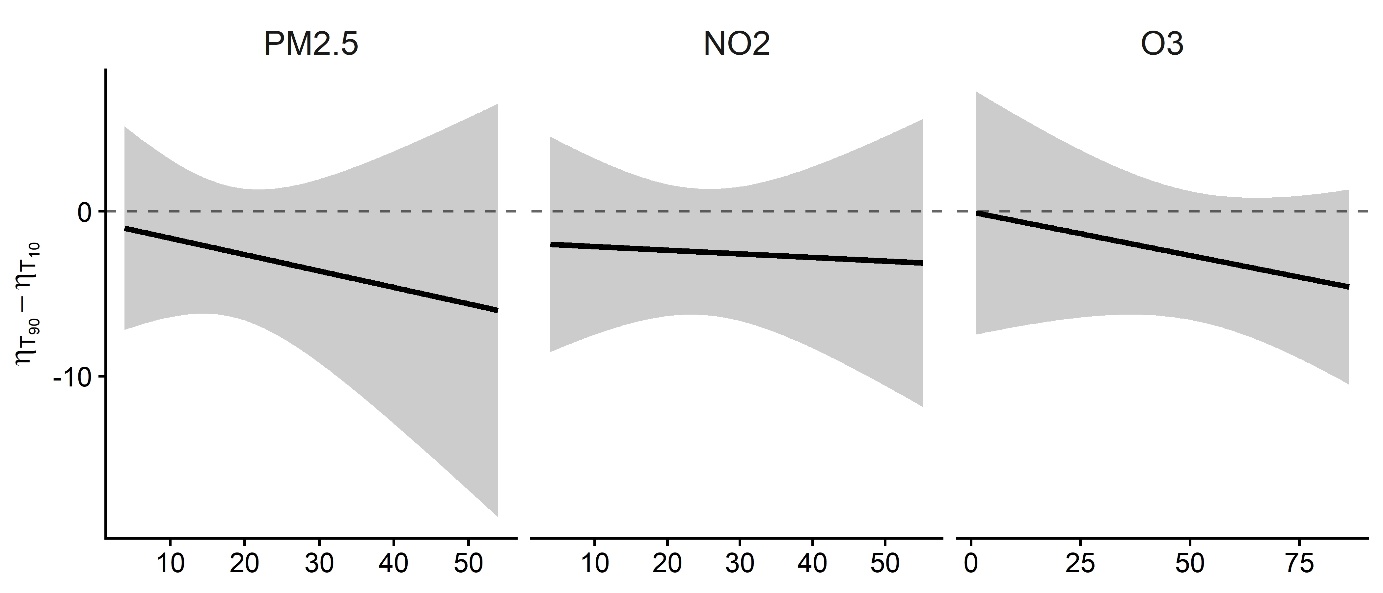


Figure S5: Estimated difference in the linear predictor comparing the 90^th^ versus 10^th^ percentile of temperature (η_{T90} − η_{T10}) across pollutant concentrations from generalized additive models of DBP. Curves are shown separately for PM_2.5_, NO_2_, and O₃. Shaded areas represent 95% confidence intervals.

**a)**


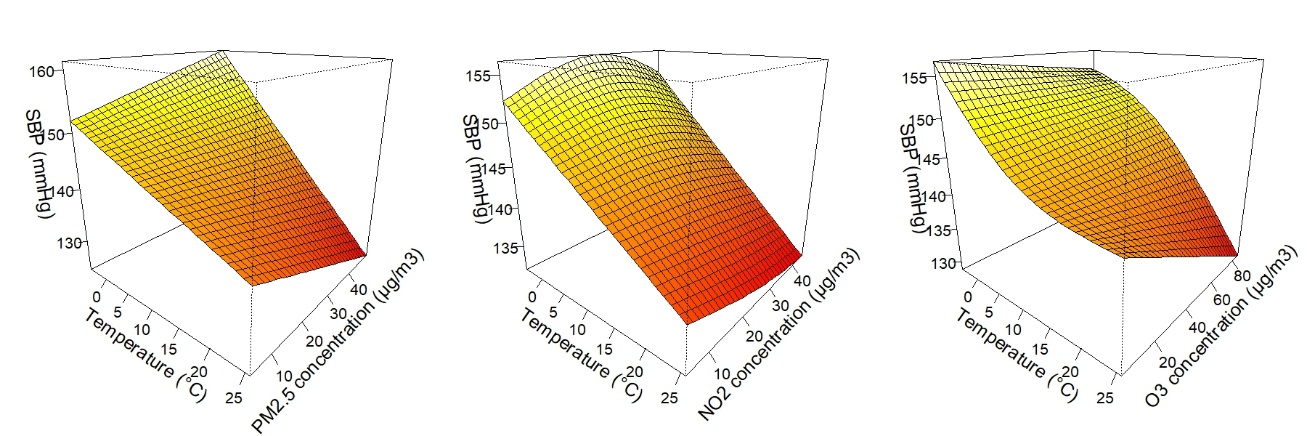


**b)**


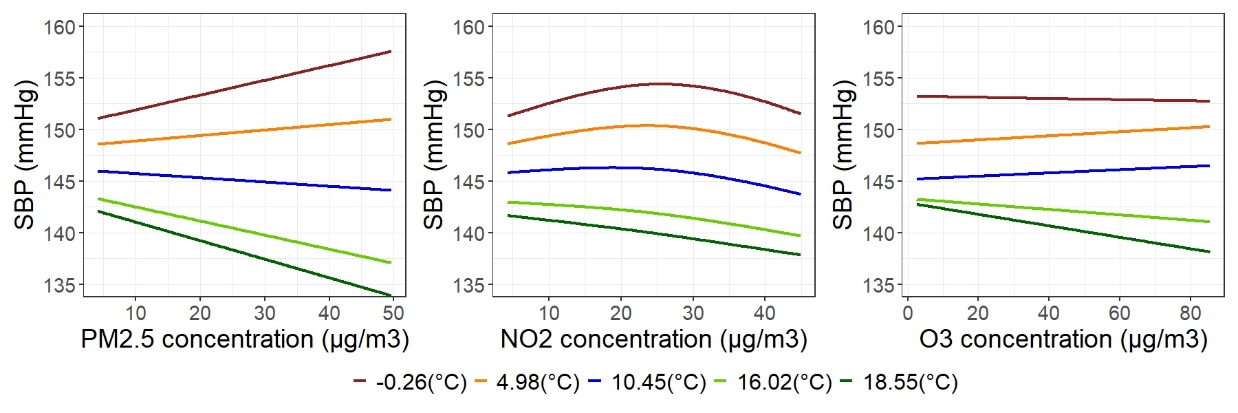


Figure S6: Lag 0–1-day associations between temperature, air pollution, and SBP. (*Panel* **a**) Visual representation of the bivariate response surfaces for SBP of temperature and air pollutants, PM_2.5_, NO_2_, and O_3_ (*column-wise*), estimated by the tensor product model. All models were adjusted for age, body mass index (BMI), socioeconomic status (SES), location (urban/rural), packyears, current smoking (yes/no), second smoker (yes/no), fossil fuel heating (yes/no), the season, and relative humidity (binary with cutoff 80%). The color intensity represents the magnitude of the BP values. Darker colors indicate lower values; lighter colors represent larger values. (*Panel* **b**) The adjusted associations between SBP and air pollutants at the 10th, 25th, 50th, 75th, and 90th percentiles of the Tmean distribution.

**a)**


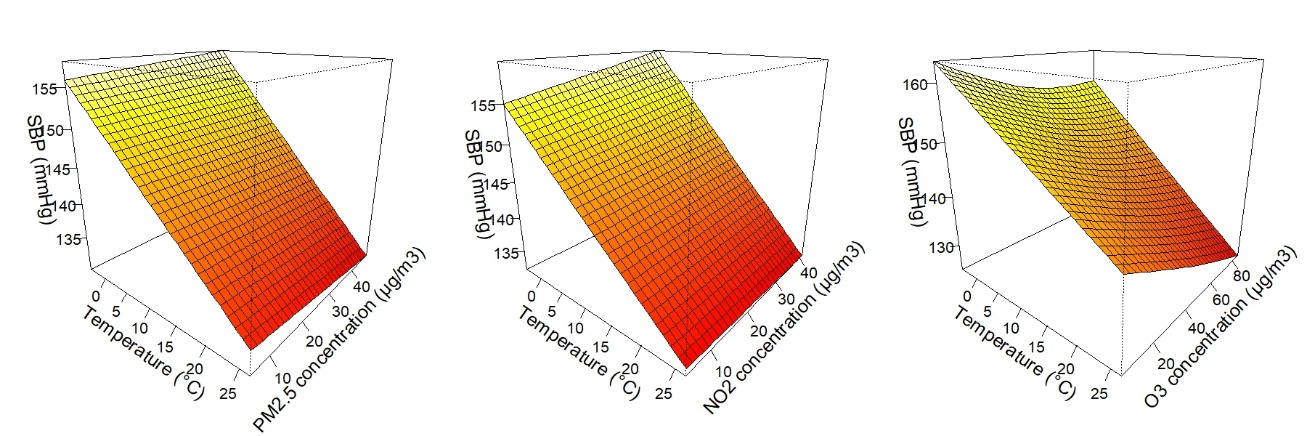


**b)**


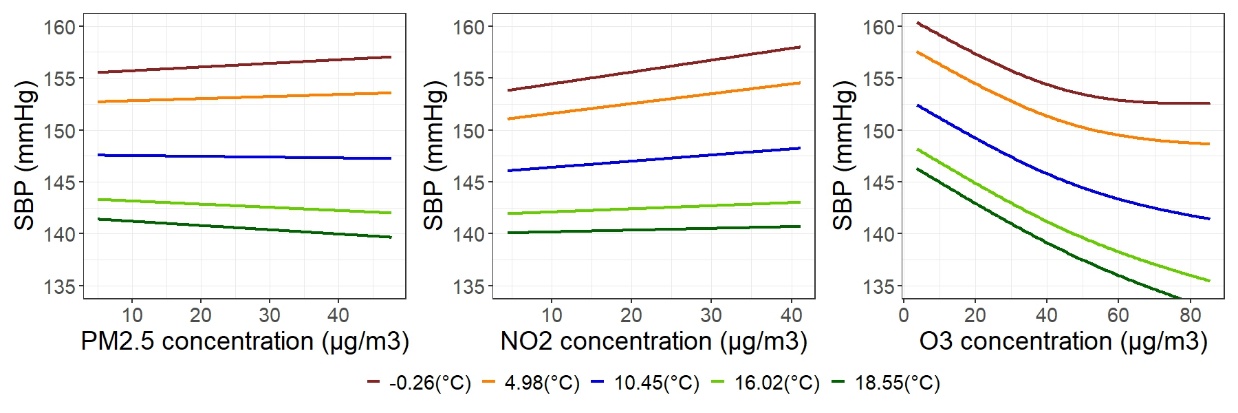


Figure S7: Lag 0–3-day associations between temperature, air pollution, and SBP. (*Panel* **a**) Visual representation of the bivariate response surfaces for SBP of temperature and air pollutants, PM_2.5_, NO_2_, and O_3_ (*column-wise*), estimated by the tensor product model. All models were adjusted for age, body mass index (BMI), socioeconomic status (SES), location (urban/rural), packyears, current smoking (yes/no), second smoker (yes/no), fossil fuel heating (yes/no), the season, and relative humidity (binary with cutoff 80%). The color intensity represents the magnitude of the BP values. Darker colors indicate lower values; lighter colors represent larger values. (*Panel* **b**) The adjusted associations between SBP and air pollutants at the 10th, 25th, 50th, 75th, and 90th percentiles of the Tmean distribution.

**a)**


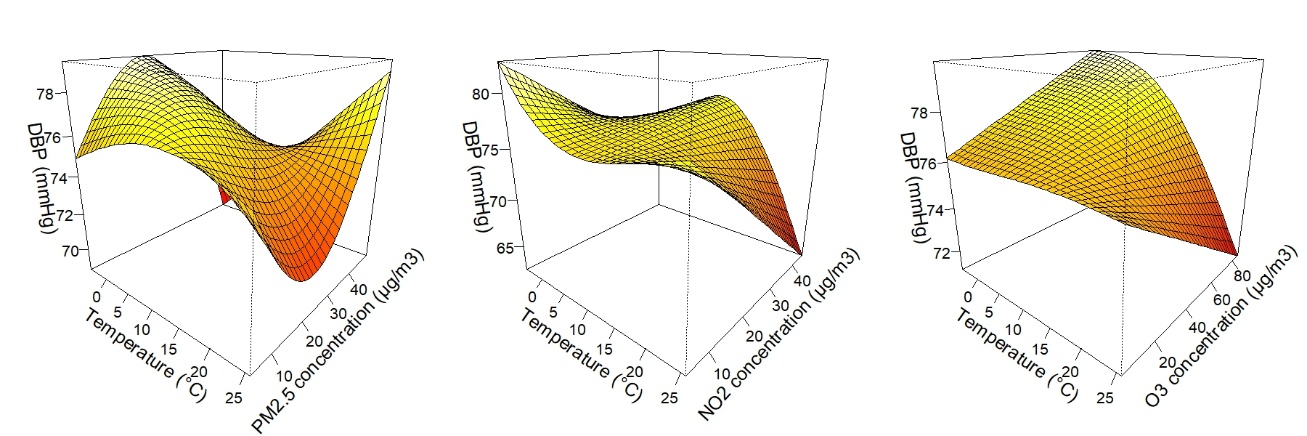


**b)**


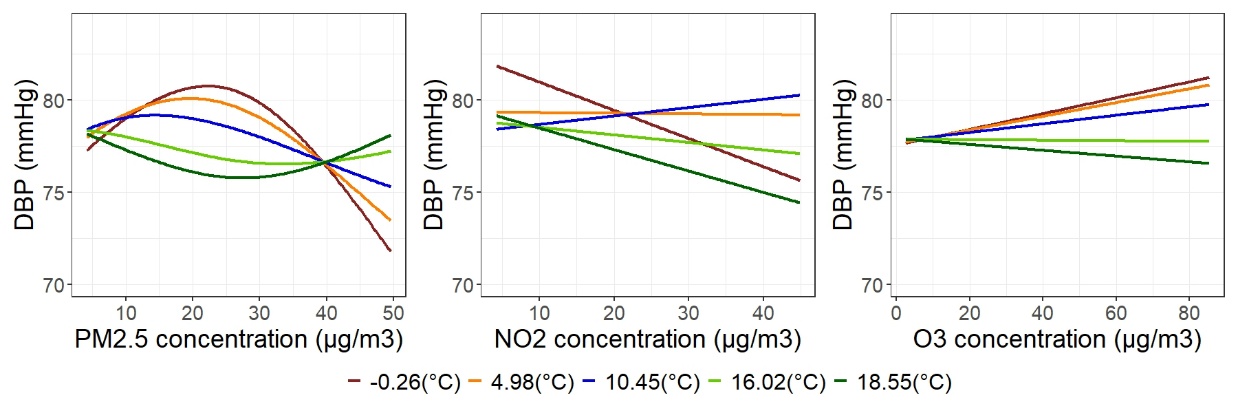


Figure S8: Lag 0–1-day associations between temperature, air pollution, and DBP. (*Panel* **a**) Visual representation of the bivariate response surfaces for SBP of temperature and air pollutants, PM_2.5_, NO_2_, and O_3_ (*column-wise*), estimated by the tensor product model. All models were adjusted for age, body mass index (BMI), socioeconomic status (SES), location (urban/rural), packyears, current smoking (yes/no), second smoker (yes/no), fossil fuel heating (yes/no), the season, and relative humidity (binary with cutoff 80%). The color intensity represents the magnitude of the BP values. Darker colors indicate lower values; lighter colors represent larger values. (*Panel* **b**) The adjusted associations between SBP and air pollutants at the 10th, 25th, 50th, 75th, and 90th percentiles of the Tmean distribution.

**a)**


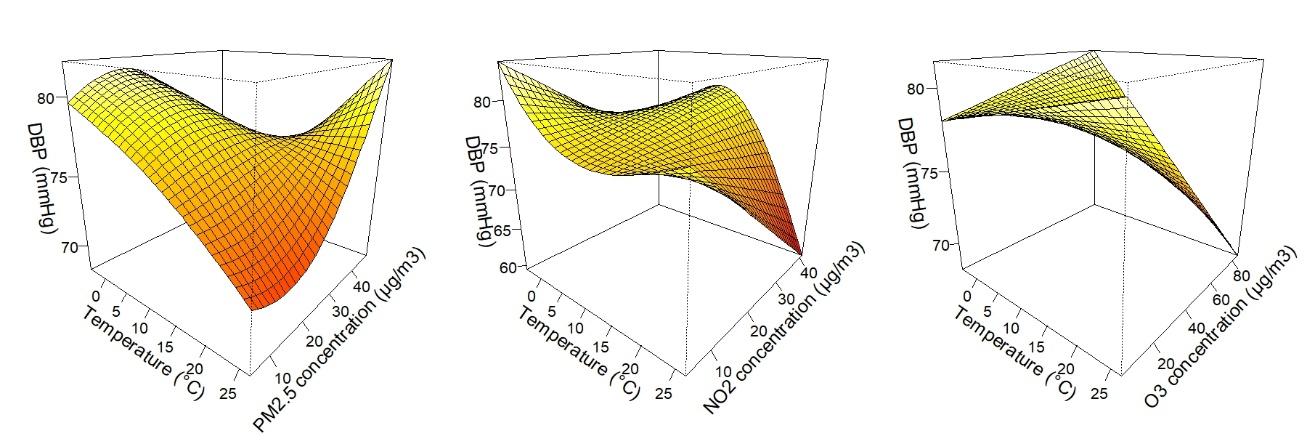


**b)**


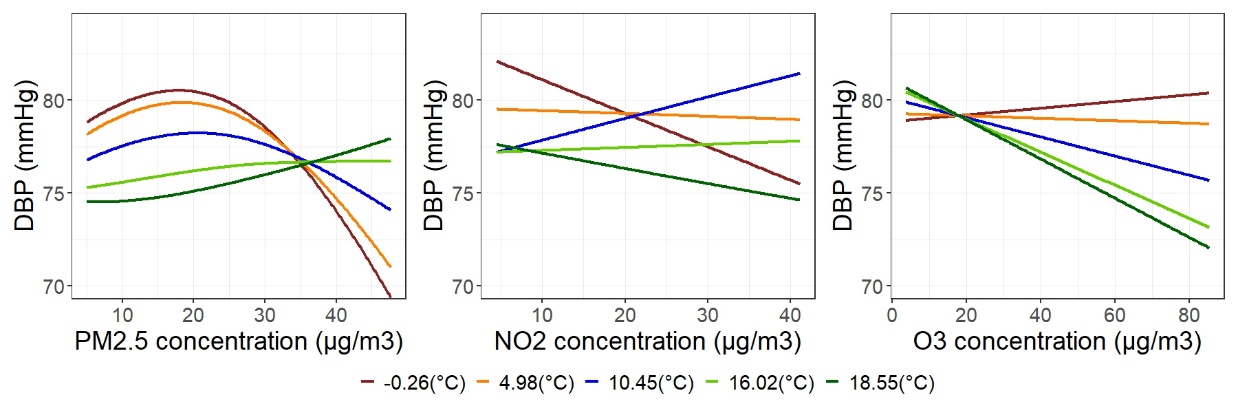


Figure S9: Lag 0–3-day associations between temperature, air pollution, and DBP. (*Panel* **a**) Visual representation of the bivariate response surfaces for SBP of temperature and air pollutants, PM_2.5_, NO_2_, and O_3_ (*column-wise*), estimated by the tensor product model. All models were adjusted for age, body mass index (BMI), socioeconomic status (SES), location (urban/rural), packyears, current smoking (yes/no), second smoker (yes/no), fossil fuel heating (yes/no), the season, and relative humidity (binary with cutoff 80%). The color intensity represents the magnitude of the BP values. Darker colors indicate lower values; lighter colors represent larger values. (*Panel* **b**) The adjusted associations between SBP and air pollutants at the 10th, 25th, 50th, 75th, and 90th percentiles of the Tmean distribution.
